# Supplementary material for: Water and heat exchanges in mammalian lungs
Source: Sci Rep. 2023 Apr 24;13:6636. doi: 10.1038/s41598-023-33052-y (PMC10126058; doi:10.1038/s41598-023-33052-y)
Supplement: Supplementary file 1 — Supplementary Information. [file 41598_2023_33052_MOESM1_ESM.pdf]

Water and heat exchanges in mammalian lungs - Supplementary material

Benoit Haut, Cyril Karamaoun, Benjamin Mauroy, Benjamin Sobac

1 Péclet number in the bronchial region of mammals

Figures S1.a and S1.b give the Péclet number Pe_i (Equation 1 of the paper) as a function of the generation index i and for mammals of different sizes, at rest (Figure S1.a) and at maximal effort (Figure S1.b). Figures S1.c and S1.d give the key dimensionless number Pe_i/β^2 (Equation 2 of the paper) as a function of the generation index i and for mammals of different sizes, at rest (Figure S1.c) and at maximal effort (Figure S1.d).

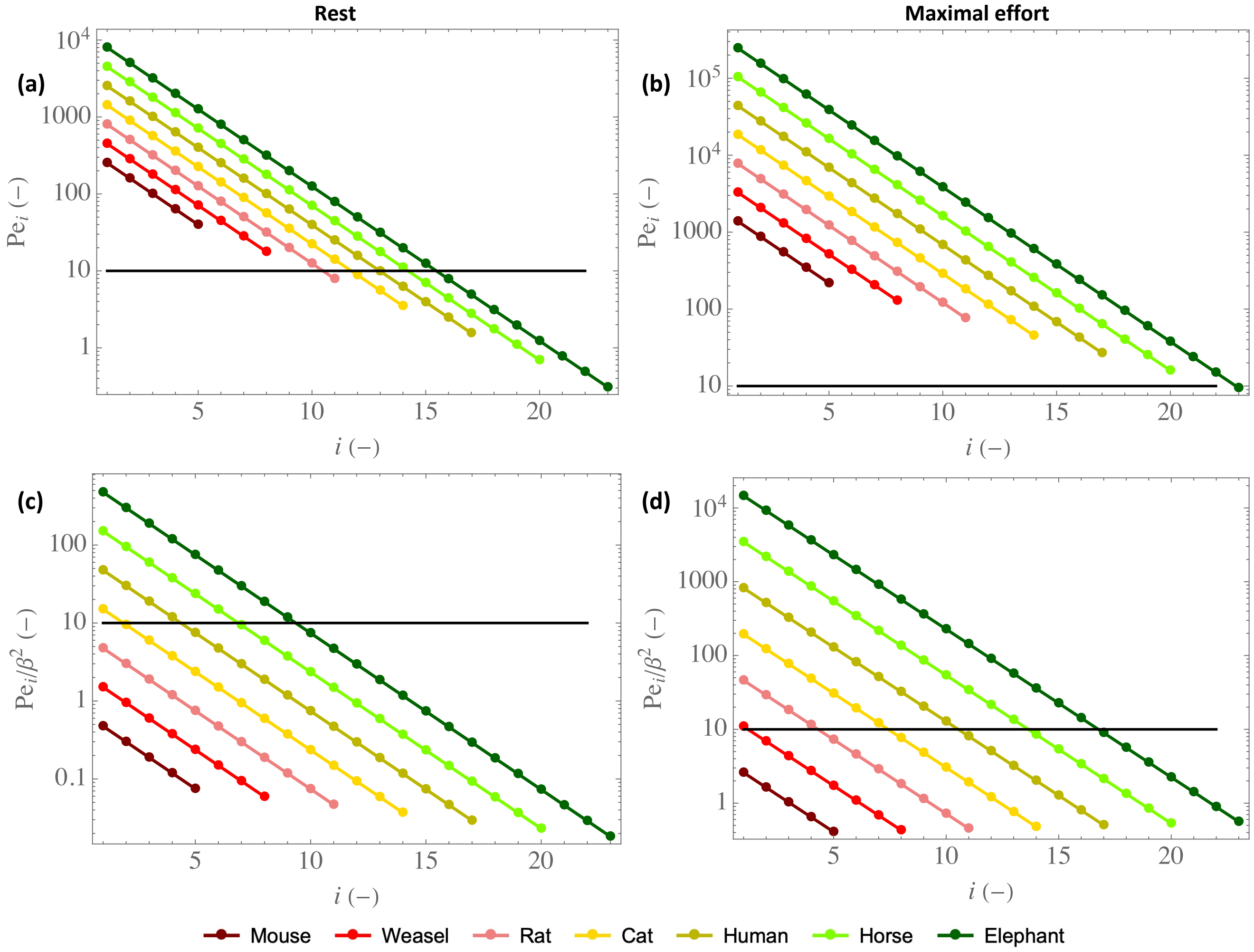

Figure S1: Péclet number Pe_i as a function of i and for mammals of different sizes, at rest **(a)** and at maximal effort **(b)**. Pe_i/β^2 as a function of i and for mammals of different sizes, at rest **(c)** and at maximal effort **(d)**. "Mouse" corresponds to $M = 5$ g, "Weasel" corresponds to $M = 50$ g, "Rat" to $M = 500$ g, "Cat" to $M = 5$ kg, "Human" to $M = 50$ kg, "Horse" to $M = 500$ kg and "Elephant" to $M = 5000$ kg. These figures have been established using the data presented in Tables 1 and 2 of the paper.

2 Numerical simulations of the water vapor and momentum transports in the lumen of an airway

First of all, note that all the assumptions regarding the modeling of the transports in the lumen made in the paper (stationary-state, axial symmetry...) are also assumed to be applicable in this supplementary material. Moreover, please remind that a sketch of an airway, introducing several parameters as well as the different phenomena involved, is provided by Figure 1.d of the paper.

In order to analyze in detail the water vapor and momentum transports in the lumen of an airway in generation i, we complete Equation 3 of the paper (water vapor transport in the lumen of the airway) with the dimensionless continuity equation and, under a lubrification approximation as the flow in the airway is mainly directed in the z direction, the dimensionless momentum (along z and r) balance equations:

$$\frac{1}{r}\frac{\partial}{\partial r}(rv_r) + \frac{1}{\beta}\frac{\partial v_z}{\partial z} = 0 \tag{S1}$$

$$v_r\frac{\partial v_z}{\partial r} + \frac{1}{\beta}v_z\frac{\partial v_z}{\partial z} = -\frac{1}{\beta}\frac{\partial p}{\partial z} + \frac{\beta\,\mathrm{Sc}}{\mathrm{Pe}_i}\frac{1}{r}\frac{\partial}{\partial r}\left(r\frac{\partial v_z}{\partial r}\right) \tag{S2}$$

$$\frac{\partial p}{\partial r} = 0 \tag{S3}$$

where $\mathrm{Sc} = \nu/\mathcal{D}$ is Schmidt number of the air, with ν its kinematic viscosity, and p is the dimensionless pressure.

These equations are completed by boundary conditions. At the center of the airway ($\forall z \in]0,1[$, $r = 0$), the assumption of axial symmetry gives:

$$v_r = 0\ ,\ \frac{\partial v_z}{\partial r} = 0 \tag{S4}$$

As mentioned in the paper, a no-slip condition is used for the air velocity at the lumen–ASL interface ($\forall z \in]0,1[$, $r = 1$):

$$v_r = v_z = 0 \tag{S5}$$

Finally, at the beginning of the airway ($\forall r \in [0,1]$, $z = 0$), we assume a “flat” velocity profile:

$$v_z = 1 \text{ and } v_r = 0 \tag{S6}$$

This “flat” velocity profile is motivated by the fact that the bifurcations in the bronchial tree create recirculations of the flow. This results in a non-established velocity profile at the entrance of the airway (i.e. differing significantly from a “Poiseuille” parabolic velocity profile). Moreover, the flow-establishment length may be non negligible in front of the length of the airway (what is sketched in Figure 1.d of the paper), or even way larger in proximal generations. This causes a strong increase of the transfers in the airways [2] and previous works have shown that considering a constant axial velocity at the entrance of the airway allows a good estimation of this enhancement of the transfers [2, 3].

The solution of Equations S1-S6 gives the velocity field in the lumen of the airway. Thus, the subsequent solving of Equation 3 in the paper (with its boundary conditions $c_i(z,1) = c_{\mu,i}$ and $\partial c_i/\partial r|_{r=0} = 0$) gives the water vapor concentration field in this lumen. To solve these equations, a numerical procedure was established in Wolfram Mathematica 12, using a finite difference method. Second order upwind schemes are used for the convective terms, while the diffusion terms are discretized using a second order centered scheme. A first order scheme is used to discretize the pressure gradient. The mesh independency of the results has been checked. For this purpose, 75 discretization points are used in the z direction and 150 points are used in the r direction.

To mimic the fact that the recirculations at bifurcations tend to homogenize the water vapor concentration field at the entrance of the airway, let us consider the case of a uniform concentration field at this entrance (i.e. $c_i(0,r) = c_0$). Moreover, let us rescale the vapor concentration field as $\mathcal{C}_i(z,r) = (c_i(z,r) - c_0)/(c_{\mu,i} - c_0)$ (i.e. the rescaled concentration is equal to 0 at the entrance of the airway and equal to 1 at the lumen–ASL interface). In Figure S2, contour plots of the rescaled concentration field $\mathcal{C}_i(z,r)$, obtained by the numerical solving of Equations S1-S6 and Equation 3 of the paper (with its boundary conditions) are presented for $\beta = 7$ ($M = 70$ kg) and three different values of Pe/β^2: $\mathrm{Pe}/\beta^2 = 1$, $\mathrm{Pe}/\beta^2 = 10$ and $\mathrm{Pe}/\beta^2 = 100$.

Equations S1-S6 and Equation 3 of the paper (with its boundary conditions) show that Sh_i is a sole function of β and Pe_i (and Sc, but it is a constant in our problem as we evaluate the properties of the air at the body temperature and at a relative humidity of 100%). In order to construct a correlation linking Sh_i to β and Pe_i,

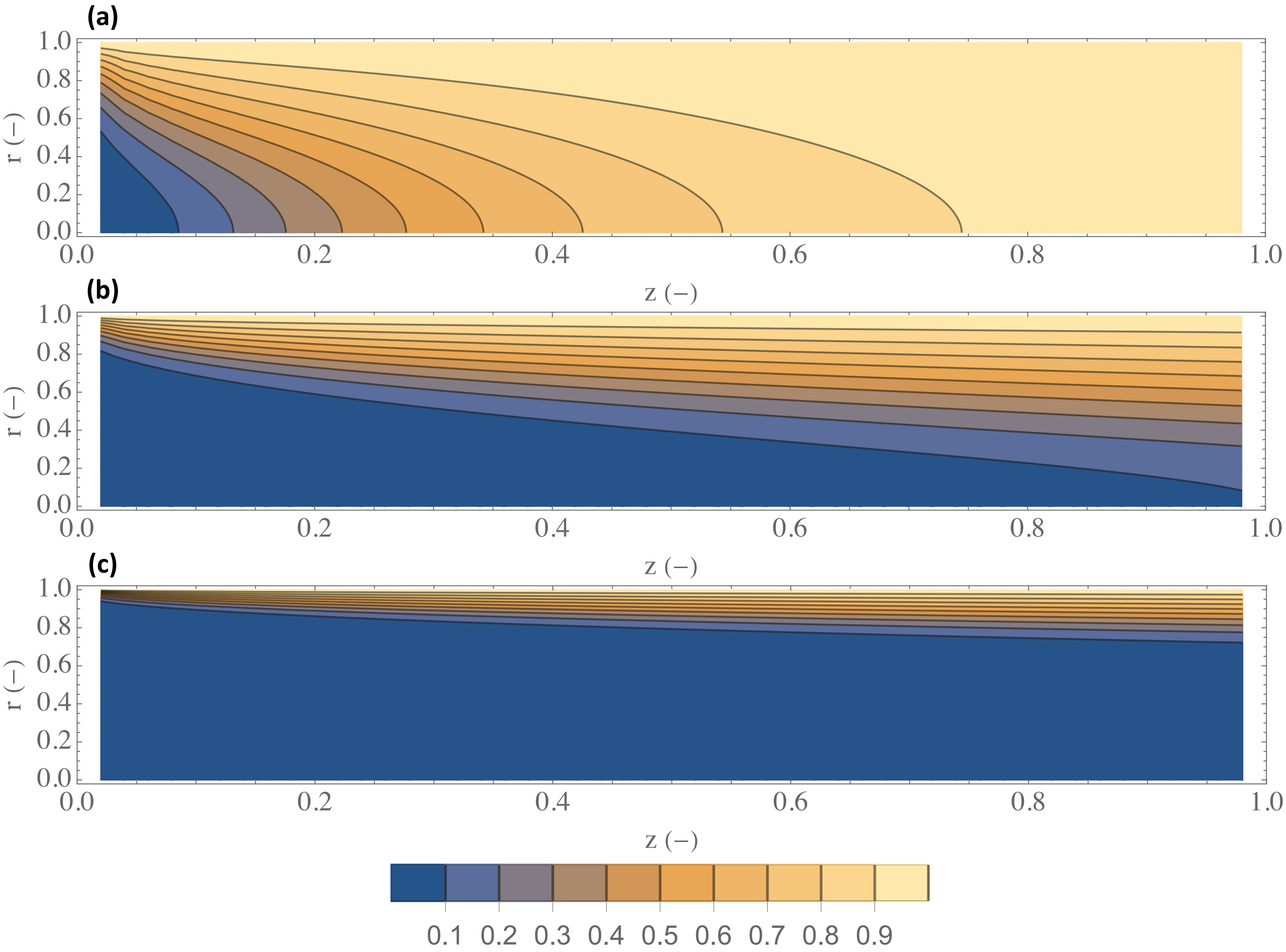

Figure S2: Contour plots of the rescaled concentration field $\mathcal{C}_i(z, r)$, for $\beta = 7$ and three values of Pe/β^2: **(a)** $\mathrm{Pe}/\beta^2 = 1$, **(b)** $\mathrm{Pe}/\beta^2 = 10$ and **(c)** $\mathrm{Pe}/\beta^2 = 100$.

these equations have been solved for different values of β and Pe_i, and with the rescaled concentration field. For given values of β and Pe_i, after solving the equations, Sh_i was evaluated using the following equation (equivalent to Equation 7 of the paper, but for the rescaled concentration field):

$$\mathrm{Sh}_i = -\mathrm{Pe}_i \frac{\log\left(1 - \bar{\mathcal{C}}_i(1)\right)}{2\beta^2} \tag{S7}$$

with $\bar{\mathcal{C}}_i(1)$ the velocity-averaged rescaled water vapor concentration at $z = 1$ (see Equation 5 of the paper).

Figure S3 presents Sh_i as a function of Pe_i/β^2, for several values of β. The interval considered for β (i.e. $4 < \beta < 23$) corresponds to values of the mass between 5 g and 5000 kg (see Table 1 of the paper). For a given value of β, which corresponds to a given value of the mass M, the lower limit considered for Pe_i is the maximum between 10 and the Péclet number at rest in generation n of a mammal of this mass, while the upper limit is the Péclet number in the trachea of this mammal, at maximal effort. Note that there is no physical meaning to characterize Sherwood for Péclet smaller than 10 with the equations used in this paper as we neglect the axial diffusive transport in the lumen, which is not small in front of the convective one for such low Péclet.

The results presented in Figure S3 show that Sh_i appears to be really well correlated with Pe_i/β^2, and that this correlation can be fitted by the following equation:

$$\mathrm{Sh}_i = 1.3 + \sqrt{\frac{\mathrm{Pe}_i}{2\beta^2}} \tag{S8}$$

Please remind that this correlation should not be used for $\mathrm{Pe}_i < 10$.

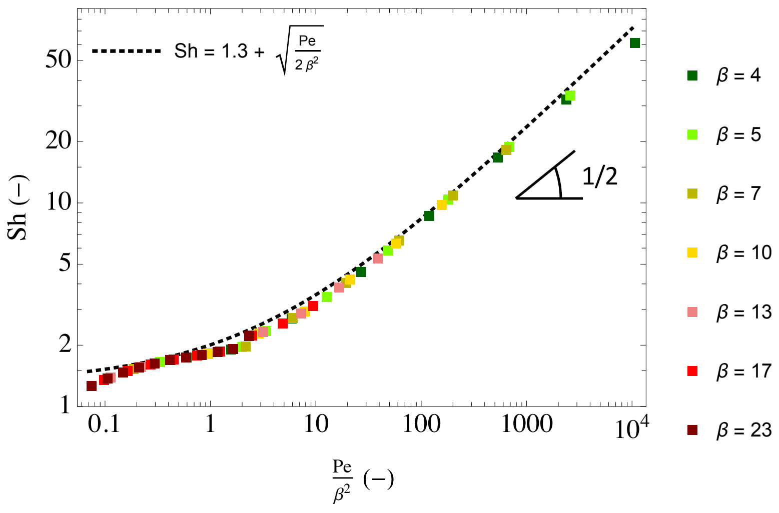

Figure S3: Sh_i as a function of Pe_i/β^2, for several values of β. The squares are results of the numerical simulations of Equations S1-S6 and Equation 3 of the paper (and its boundary conditions). The dashed curve is given by Equation S8.

Note that this correlation shows that, when $\mathrm{Pe}_i/\beta^2 \gg 1$, $\mathrm{Sh}_i \propto \sqrt{\mathrm{Pe}_i/(\beta^2)}$. This can be understood easily by noting that, when $\mathrm{Pe}_i/\beta^2 \gg 1$, the diffusion boundary layer (i.e. the concentration gradients) is located near the ASL–lumen interface in the entire airway (situation presented in Figure S2.c). Hence, the thickness of this boundary layer at the dimensionless position z in the airway is $\delta_i(z) \simeq \sqrt{2\mathcal{D}zL_i/U_i}$, with $\mathcal{D}$ the diffusion coefficient of water vapor in air, U_i the average axial velocity of the air in the airway and L_i the length of the airway. Consequently, the mass transfer coefficient k_i between the ASL–lumen interface and the airway can be approximated as $k_i \simeq \mathcal{D}/\int_0^1 \delta_i(z)dz = \frac{3}{2}\sqrt{\mathcal{D}U_i/(2L_i)}$. As $\mathrm{Sh}_i = k_iR_i/\mathcal{D}$, with R_i the radius of the airway, and $\mathrm{Pe}_i = U_iL_i/\mathcal{D}$, it gives $\mathrm{Sh}_i \simeq \frac{3}{2}\sqrt{\mathrm{Pe}_i/(2\beta^2)}$, with $\beta = L_i/R_i$.

Finally, it worth to mention that the flow in the trachea and in the few generations downstream can be turbulent, especially at high inspiration and expiration flow rates and for large mammals. This feature is not included in our model as only steady states are considered, but it has limited impact on the Sherwood number. Indeed, as mentioned previously, in these proximal generations of the lungs, the flow-establishment length is usually significantly larger than the length of the airways, and the undeveloped character of the flow in these proximal airways has more impact on the transfers than the potentially turbulent nature of the flow [1, 2]. As mentioned above, the simulations of the momentum and mass transport equations in a single airway, used to construct the correlation of Sh, account for this undeveloped character of the flow by imposing a constant axial velocity at the inlet of an airway (Equation S6). Moreover, the results show that, especially at maximal effort, the proximal generations of the lungs hardly contribute to the air conditioning (see the Results section of the paper). Hence, a precise evaluation of Sh in the first generations of the lungs might not be necessary to give an accurate view of the air conditioning in the lungs at maximal effort.

3 Analytical approximation of the location of maximal extraction

To obtain an analytical approximation of $i_{\max}$, the index of the generation in which the maximal amount of water (or heat) is extracted, we place ourself in a simplified situation in which the ASL is maintained at body temperature (i.e. $c_{\mu,i} = c_{\mathrm{sat}}(T_b)$). In this situation, the balance equation during inspiration (Equation 11 of the paper) becomes:

$$c_i^{\mathrm{insp}} = c_{i-1}^{\mathrm{insp}} + \Gamma_i(c_{\mathrm{sat}}(T_b) - c_i^{\mathrm{insp}}) \tag{S9}$$

or:

$$c_{\mathrm{sat}}(T_b) - c_i^{\mathrm{insp}} = \frac{c_{\mathrm{sat}}(T_b) - c_{i-1}^{\mathrm{insp}}}{1 + \Gamma_i} \tag{S10}$$

Hence:

$$c_{\mathrm{sat}}(T_b) - c_i^{\mathrm{insp}} = \frac{c_{\mathrm{sat}}(T_b) - c_0^{\mathrm{insp}}}{\Pi_{j=1}^{j=i}(1 + \Gamma_j)} \tag{S11}$$

and:

$$c_i^{\text{insp}} - c_{i-1}^{\text{insp}} = \Gamma_i (c_{\text{sat}}(T_b) - c_i^{\text{insp}}) = \frac{\Gamma_i}{\Pi_{j=1}^{j=i}(1+\Gamma_j)} (c_{\text{sat}}(T_b) - c_0^{\text{insp}}) \tag{S12}$$

As the amount of water extracted per unit of time from the ASL of all the airways in generation i ($E_{w,i}$) is $\propto$ $c_i^{\text{insp}} - c_{i-1}^{\text{insp}}$, we can write that:

$$\frac{E_{w,i}}{E_{w,i-1}} = \frac{\Gamma_i}{\Gamma_{i-1}} \frac{1}{1+\Gamma_i} \tag{S13}$$

Now, we replace Γ_i by its approximate expression $\Gamma_{i,\text{lim}} = \sqrt{2\beta^2/\text{Pe}_i}$ (Equation 19 of the paper), which is a good approximation if $\text{Pe}_i/\beta^2 \gg 1$. Moreover, we see, with Equation 1 of the paper, that $\Gamma_{i,\text{lim}}/\Gamma_{i-1,\text{lim}} = \sqrt{\text{Pe}_{i-1}/\text{Pe}_i} = \sqrt{2h} = 2^{1/3}$ (as $h = 2^{-1/3}$). Thus, as $i = i_{\max} \Leftrightarrow E_{w,i} = E_{w,i-1}$, $i_{\max}$ satisfies the following equation:

$$\sqrt{\frac{2\beta^2}{\text{Pe}_{i_{\max}}}} = 2^{\frac{1}{3}} - 1 \tag{S14}$$

Using Equation 2 of the paper, the previous equation can be rewritten as:

$$2^{\frac{i_{\max}-1}{6}} = \left(2^{\frac{1}{3}} - 1\right) \sqrt{\frac{\text{Pe}_{1,\text{ref}}}{2\beta_{\text{ref}}^2}} \sqrt{\psi} \left(\frac{M}{M_{\text{ref}}}\right)^{\frac{1}{4}} \tag{S15}$$

This means that we can write that:

$$i_{\max} = \max\left(1, a + \frac{1.5\log(M) + 3\log(\psi)}{\log(2)}\right) \tag{S16}$$

with a a constant and $\max(x, y)$ the maximum of x and y.

4 Analytical approximation of the effectivity of extraction

To obtain an analytical approximation of the local effectivity of water (or heat) extraction η_i (defined in Equation 27 of the paper), we rewrite the equations of the model (Equations 11, 12 and 16 of the paper) as follows:

$$c_i^{\text{insp}} = c_{i-1}^{\text{insp}} + \Gamma_i (c_{\mu,i} - c_i^{\text{insp}}) \tag{S17}$$

$$c_{i-1}^{\text{exp}} = c_i^{\text{exp}} + \Gamma_i (c_{\mu,i} - c_i^{\text{exp}}) \tag{S18}$$

$$\Lambda_{\text{lim}}(c_{\text{sat}}(T_b) - c_{\mu,i}) = c_{\mu,i} - \frac{c_i^{\text{insp}} + c_i^{\text{exp}}}{2} \tag{S19}$$

So, as compared to the model, we consider $\Lambda_i = \Lambda_{\text{lim}}$ (given by Equation 21 of the paper), which is a good approximation if $\text{Pe}_i/\beta^2 \gg 1$, we change c_{i-1}^{exp} into c_i^{exp} in the driving force multiplying Γ_i in Equation 12 of the paper (i.e. the driving force for condensation is evaluated at the distal extremity of the generation during expiration, instead of at the proximal one), and we express $[c]_i$ in Equation 16 of the paper as $[c]_i = \frac{c_i^{\text{insp}} + c_i^{\text{exp}}}{2}$ instead of $[c]_i = \frac{c_i^{\text{insp}} + c_{i-1}^{\text{insp}} + c_i^{\text{exp}} + c_{i-1}^{\text{exp}}}{4}$.

Eliminating $c_{\mu,i}$, these equations can be rewritten as:

$$2(1+\Lambda_{\text{lim}}) \frac{c_{i-1}^{\text{insp}} - c_i^{\text{insp}}}{\Gamma_i} = -2\Lambda_{\text{lim}} c_{\text{sat}}(T_b) + (1 + 2\Lambda_{\text{lim}}) c_i^{\text{insp}} - c_i^{\text{exp}} \tag{S20}$$

$$2(1+\Lambda_{\text{lim}}) \frac{c_{i-1}^{\text{exp}} - c_i^{\text{exp}}}{\Gamma_i} = 2\Lambda_{\text{lim}} c_{\text{sat}}(T_b) + c_i^{\text{insp}} - (1 + 2\Lambda_{\text{lim}}) c_i^{\text{exp}} \tag{S21}$$

We define:

$$f_i^{\text{insp}} = 2(1+\Lambda_{\text{lim}}) \frac{c_i^{\text{insp}} - c_{i-1}^{\text{insp}}}{\Gamma_i} \tag{S22}$$

$$f_i^{\exp} = 2(1+\Lambda_{\lim})\frac{c_i^{\exp} - c_{i-1}^{\exp}}{\Gamma_i} \tag{S23}$$

Therefore:

$$-f_i^{\text{insp}} = -2\Lambda_{\lim} c_{\text{sat}}(T_b) + (1+2\Lambda_{\lim})c_i^{\text{insp}} - c_i^{\exp} \tag{S24}$$

$$-f_i^{\exp} = 2\Lambda_{\lim} c_{\text{sat}}(T_b) + c_i^{\text{insp}} - (1+2\Lambda_{\lim})c_i^{\exp} \tag{S25}$$

and:

$$-f_{i-1}^{\text{insp}} = -2\Lambda_{\lim} c_{\text{sat}}(T_b) + (1+2\Lambda_{\lim})c_{i-1}^{\text{insp}} - c_{i-1}^{\exp} \tag{S26}$$

$$-f_{i-1}^{\exp} = 2\Lambda_{\lim} c_{\text{sat}}(T_b) + c_{i-1}^{\text{insp}} - (1+2\Lambda_{\lim})c_{i-1}^{\exp} \tag{S27}$$

Thus:

$$-f_i^{\text{insp}} + f_{i-1}^{\text{insp}} = \frac{\Gamma_i}{2(1+\Lambda_{\lim})}\left((1+2\Lambda_{\lim})f_i^{\text{insp}} - f_i^{\exp}\right) \tag{S28}$$

$$-f_i^{\exp} + f_{i-1}^{\exp} = \frac{\Gamma_i}{2(1+\Lambda_{\lim})}\left(f_i^{\text{insp}} - (1+2\Lambda_{\lim})f_i^{\exp}\right) \tag{S29}$$

With this, we can see that $(f_i^{\text{insp}}, f_i^{\exp})^t$ can be expressed in the following form:

$$\begin{pmatrix} f_i^{\text{insp}} \\ f_i^{\exp} \end{pmatrix} = \kappa_i \begin{pmatrix} g_1 \\ g_2 \end{pmatrix} \tag{S30}$$

if $(g_1, g_2)^t$ is an eigenvector of the following matrix:

$$\begin{pmatrix} 1+2\Lambda_{\lim} & -1 \\ 1 & -(1+\Lambda_{\lim}) \end{pmatrix} \tag{S31}$$

Indeed, if so, a recurrence relation for κ_i can be obtained from Equations S28 and S29:

$$\kappa_{i-1} = \left(\frac{\Gamma_i}{2(1+\Lambda_{\lim})}\lambda + 1\right)\kappa_i \tag{S32}$$

with λ the eigenvalue associated with the eigenvector $(g_1, g_2)^t$ (both depending solely on $\Lambda_{\lim}$).

η_i is equal to $1 - \frac{f_i^{\exp}}{f_i^{\text{insp}}}$ and hence to $1 - \frac{g_2}{g_1}$. Therefore, evaluating the eigenvectors of matrix S31, we obtain:

$$\eta_i = 2\sqrt{\Lambda_{\lim}(1+\Lambda_{\lim})} - 2\Lambda_{\lim} \tag{S33}$$

Finally, using Equation 21 of the paper, the last equation can be rewritten as:

$$\eta_i = 2\Theta\left(\sqrt{\frac{1}{\Theta}\sqrt{\frac{\phi}{\psi}} + \frac{\phi}{\psi}} - \sqrt{\frac{\phi}{\psi}}\right) \tag{S34}$$

with Θ a constant, given by Equation 22 of the paper, and equal to 0.37 if we use the data in Tables 1 and 2 of the paper.

5 Analytical approximation of the evaporation rate in the trachea

In this section, our objective is to obtain an analytical approximation of the average, over a whole respiratory cycle, evaporation rate in the trachea J_1. It is the ratio of the amount of water extracted per unit of time from the ASL in the trachea $E_{w,1}$ (Equation 24 of the paper) to the area of the ASL–lumen interface in the trachea $2\pi R_1 L_1$:

$$J_1 = \frac{E_{w,1}}{2\pi R_1 L_1} = \frac{Q}{4\pi}\frac{c_1^{\text{insp}} - c_0^{\text{insp}} + c_0^{\exp} - c_1^{\exp}}{\beta R_1^2} \tag{S35}$$

where we have used $L_1 = \beta R_1$.

To obtain an analytical approximation of J_1, we start again with Equations S17-S19. Within this framework, the local effectivity of water extraction η_i, given by Equation S34, is independent of the generation index i. Hence,

the overall effectivity of extraction η is also given by Equation S34: $\eta = \eta_i$, $\forall i$. Therefore, using the definitions of η_i and η (Equations 27 and 28 of the paper), we can write that:

$$\eta_1 = \frac{c_1^{\text{insp}} - c_0^{\text{insp}} + c_0^{\text{exp}} - c_1^{\text{exp}}}{c_1^{\text{insp}} - c_0^{\text{insp}}} = \eta = \frac{c_0^{\text{exp}} - c_0^{\text{insp}}}{c_{\text{sat}}(T_b) - c_0^{\text{insp}}} \tag{S36}$$

These equations can be rearranged to express c_0^{exp} and c_1^{exp} as functions of η, c_0^{insp} and c_1^{insp}:

$$c_0^{\text{exp}} = c_0^{\text{insp}} + \eta(c_{\text{sat}}(T_b) - c_0^{\text{insp}}) \tag{S37}$$

$$c_1^{\text{exp}} = c_1^{\text{insp}} + \eta(c_{\text{sat}}(T_b) - c_1^{\text{insp}}) \tag{S38}$$

Moreover, we can write Equations S17 and S18 for $i = 1$:

$$c_1^{\text{insp}} = c_0^{\text{insp}} + \Gamma_1(c_{\mu,1} - c_1^{\text{insp}}) \tag{S39}$$

$$c_0^{\text{exp}} = c_1^{\text{exp}} + \Gamma_1(c_{\mu,1} - c_1^{\text{exp}}) \tag{S40}$$

The four last equations can be manipulated to eliminate c_0^{exp}, c_1^{exp} and $c_{\mu,1}$, giving:

$$c_1^{\text{insp}} - c_0^{\text{insp}} = \frac{\eta\Gamma_1}{2 - \eta(1 - \Gamma_1)}(c_{\text{sat}}(T_b) - c_0^{\text{insp}}) \tag{S41}$$

Therefore, according to Equation S36, Equation S35 can be rewritten as:

$$J_1 = \frac{Q}{4\pi}\frac{\eta^2\Gamma_1}{2 - \eta(1 - \Gamma_1)}\frac{c_{\text{sat}}(T_b) - c_0^{\text{insp}}}{\beta R_1^2} \tag{S42}$$

Now, to further develop this expression of J_1, we replace Γ_1 by its approximate expression $\Gamma_{1,\text{lim}} = \sqrt{2\beta^2/\text{Pe}_1}$ (Equation 19 of the paper), which is a good approximation for $\text{Pe}_1/\beta^2 \gg 1$. This is coherent with Equation S19, as the latter is obtained considering $\Lambda_i = \Lambda_{\text{lim}}$ (Equation 21 of the paper), which is a good approximation for $\text{Pe}_i/\beta^2 \gg 1$. As $\text{Pe}_1 = \beta Q/(\pi R_1 \mathcal{D})$ (see Equation 1 of the paper), this gives:

$$J_1 = \sqrt{\frac{Q\mathcal{D}}{8\pi\beta R_1^3}}\frac{\eta^2}{2 - \eta}(c_{\text{sat}}(T_b) - c_0^{\text{insp}}) \tag{S43}$$

To obtain this equation, we have neglected Γ_1 in front of 1, which is coherent with $\text{Pe}_1/\beta^2 \gg 1$.

This last equation allows calculating J_1 for given values of Q, β, R_1, c_0^{insp}, and of the ratio ϕ/ψ (which is needed to evaluate η, see Equation S34).

Using the allometric scalings given in Table 1 of the paper, Equation S43 can be written at rest:

$$J_1 = \sqrt{\frac{Q_{\text{ref}}\mathcal{D}}{8\pi\beta_{\text{ref}}R_{1,\text{ref}}^3}}\frac{\eta^2}{2 - \eta}(c_{\text{sat}}(T_b) - c_0^{\text{insp}})\left(\frac{M}{M_{\text{ref}}}\right)^{-\frac{1}{8}} \tag{S44}$$

and at maximal effort:

$$J_1 = \sqrt{\psi_{\text{ref}}}\sqrt{\frac{Q_{\text{ref}}\mathcal{D}}{8\pi\beta_{\text{ref}}R_{1,\text{ref}}^3}}\frac{\eta^2}{2 - \eta}(c_{\text{sat}}(T_b) - c_0^{\text{insp}})\left(\frac{M}{M_{\text{ref}}}\right)^{-\frac{1}{16}} \tag{S45}$$

Note that, as mentioned in the paper, with $\Theta = 0.37$, Equation S34 gives $\eta = 0.68$ at rest ($\phi = \psi = 1$) and $\eta = 0.57$ at maximal effort ($\phi/\psi = 0.25$ if we use the data in Table 1 of the paper).

6 Nomenclature

Roman symbols

c	Concentration of water vapor in air, $\text{mol}\,\text{m}^{-3}$
$\mathcal{C}$	Rescaled water vapor concentration field, -
$\mathcal{D}$	Diffusion coefficient of water in air, $\text{m}^2\,\text{s}^{-1}$
E_w	Amount of water extracted per unit of time, $\text{mol}\,\text{s}^{-1}$

h	Ratio of the radii of airways in two successive generations, -
i	Generation index, -
J	Evaporation rate of water, $\mathrm{mol\,m^{-2}\,s^{-1}}$
L	Length of the airway, m
M	Mass of the body, kg
n	Number of generations in the bronchial tree, -
Pe	Péclet number, -
p	Pressure in the lumen, -
Q	Inspiration flow rate, $\mathrm{m^3\,s^{-1}}$
r	Radial coordinate, -
R	Radius of the airway, m
Sc	Schmidt number of the air in the lumen, -
Sh	Sherwood number of the mass transfer between the ASL and the lumen, -
T	Temperature, K
U	Average axial velocity of the air in the airway, $\mathrm{m\,s^{-1}}$
v	Velocity of the air, -
z	Axial coordinate, -

Greek symbols

β	L/R ratio of the airway, -
δ	Thickness of the boundary layer, m
η	Effectivity of extraction, -
Λ	Dimensionless number, see Equation 17 of the paper
ν	Kinematic viscosity of the air, $\mathrm{m^2\,s^{-1}}$
ϕ	Factor accounting for the increase of the cardiac flow rate during a possible effort, -
Γ	Dimensionless number, see Equation 9 of the paper
Θ	Dimensionless constant, see Equation 22 of the paper
ψ	Factor accounting for the increase of the inspiration flow rate during an effort, -

Subscripts and superscripts

$\bar{\mathcal{C}}$	Velocity-average of the rescaled concentration field
0	At the inlet of the airway
b	Body
exp	Related to the expiration
i	In generation i
insp	Related to the inspiration
lim	Γ or Λ for $\mathrm{Pe}/\beta^2 \gg 1$
max	Maximum of extraction
r	Component along the r axis
ref	At the reference mass M_{ref}
sat	Saturated with water
z	Component along the z axis
μ	At the ASL–lumen interface

References

[1] T. J. Pedley, R. C. Schroter, and M. F. Sudlow. Energy losses and pressure drop in models of human airways. *Respiration Physiology*, 9(3):371–386, 1970.

[2] T. J. Pedley, R. C. Schroter, and M. F. Sudlow. The prediction of pressure drop and variation of resistance within the human bronchial airways. *Respiration Physiology*, 9(3):387–405, 1970.

[3] A. K. Wells, I. P. Jones, I. S. Hamill, and R. Bordas. The prediction of viscous losses and pressure drop in models of the human airways. *Respiratory Research*, 34(1):–, 2018.
